# Implications of a Remote Study of Children With Cerebral Visual Impairment for Conducting Virtual Pediatric Eye Care Research: Virtual Assessment Is Possible for Children With CVI

**DOI:** 10.3389/fnhum.2021.733179

**Published:** 2021-09-14

**Authors:** Reem Almagati, Barry S. Kran

**Affiliations:** ^1^New England College of Optometry (NECO), Boston, MA, United States; ^2^NECO Center for Eye Care at Perkins, Watertown, MA, United States

**Keywords:** cerebral visual impairments, pediatric eye research, assessment, synchronous, asynchronous

## Abstract

The Pandemic of 2020 impacted conducting in-person research. Our proposed project already had an asynchronous online component but was later morphed to add a synchronous online component, thereby eliminating the need for in-person assessment. The project compares the results of various tests between a group of children with Cerebral Visual Impairments (CVI) (*N* = 4) and an age-matched sample of children without CVI (*N* = 3) from a pediatric low vision clinic. This model was trialed with a small convenient sample of typically developing children in the same age range (*N* = 4). Given the positive feedback, recruitment for the larger study was done *via* encrypted e-mail rather than through traditional mailing. The asynchronous components included recruitment, pre-assessment information, the Flemish CVI questionnaire, Vineland-3 comprehensive parent questionnaire for assessment of age equivalent, and vision function tests, such as contrast sensitivity. The synchronous components were administered *via* Zoom telehealth provided by necoeyecare.org and included assessment of visual acuity *via* the Freiburg Visual Acuity and Contrast Test (FrACT) electronic software and assessment of visual perceptual batteries *via* the Children’s Visual Impairment Test for developmental ages 3–6-years (CVIT 3–6). Our virtual testing protocol was successful in the seven participants tested. This paper reviews and critiques the model that we utilized and discusses ways in which this model can be improved. Aside from public health considerations during the pandemic, this approach is more convenient for many families. In a broader perspective, this approach can be scaled for larger N studies of rare conditions, such as CVI without being confined by proximity to the researcher.

## Introduction

Telehealth refers to the use of digital modalities to access information which in turn allows the clinician to provide healthcare services remotely. These remote means typical fall under video calls (real in-time/synchronous audio and video communication), audio calls and asynchronous/email communication with the patient (Board on Health Care Services and Institute of Medicine, 2012). Technological advancement facilitated the development of telehealth. The first technology-assisted remote diagnosis took place in 1948 where a radiology photograph was sent *via* telephone across a distance of 24 miles (Gershon-Cohen and Cooley, 1950). The birth of the internet expanded the use of telehealth *via* creating a global communication network. Caffery et al. (2019) reviewed 78 articles of tele-ophthalmic models of eyecare. They reported that the majority of services were either for general eyecare or emergency services. The authors also demonstrated that tele-ophthalmology is feasible for screening, consultation and follow-up care of various ophthalmic conditions. The novel global acute respiratory syndrome, that result in the Coronavirus Disease of 2019 (COVID-19) pandemic, caused a spike in telehealth medical services. Telehealth services complied with the Center of Disease Control (CDC) quarantine and social distancing guidelines. Now, multiple venues/platforms are available which are compliant with Health Insurance Portability and Accountability Act (HIPPA). These include Zoom for Healthcare (©Zoom, Inc. San Jose, CA, United States), VSee (©Sunnyvale, CA, United States), and Doxyme Pro (©LLC, Salt Lake City, Utah, United States). While telehealth medicine may have some benefits over in-person delivery of medical services, in which it is convenient, cost-effective especially for routine examination, it is limited in the scope of care provided, licensing and insurance coverage. In response to the COVID-19 pandemic, human subjects research has either shutdown/paused or adapted necessary changes to accommodate safe clinical practice (Mourad et al., 2020). This paper describes adaptation of a model for both synchronous and asynchronous data collection for a pediatric eye care research project. This approach may be particularly valuable for gathering large group data for clinically significant but rare conditions, such as CVI.

## Research During the Pandemic

The current pandemic greatly impacted the research processes which pushed researchers to find innovative ways to move forward with remotely based research models while adhering to rigorous research practices. This required major protocol and procedural changes. In recruitment, written consent changed to electronically obtained consent. In addition, internet access automatically became an inclusion criterion. We had originally planned to conduct a study to evaluate visual perceptual abilities in children with cerebral visual impairments (CVI) vs. children with ocular and/or ocular-motor disorders only *via* two CVI tools (Vancleef et al., 2020). Sakki et al. (2018) defined CVI as a verifiable visual impairment which is not attributed to anterior visual pathway pathology and/or ocular disorders. However, in-person research was no longer feasible due to public health considerations during the pandemic. We developed and trialed a virtual model in a small sample of typically developing young children. The feasibility of the remote applicability of the synchronous component: the FrACT and the CVIT 3–6 and the asynchronous component: The Flemish CVI questionnaire and the Vineland-3 Comprehensive parent questionnaire was evaluated. The pilot study consisted of four participants ages 3–5 years old, success was defined as successful completion of research tasks in at least 75% of participants. Upon review of the pilot data, we determined to proceed with the same approach for the CVI study. The CVI study had a total of seven participants, four of which had a previous diagnosis of CVI. The diagnosis was made at NECO center for eye care at Perkins by Drs. Barry Kran and Nicole Ross. Caregivers provided digital copies of signed informed consent and assent in accordance with the Declaration of Helsinki. The experiment protocol was approved by the institutional review board of New England College of Optometry. Description of material and method is provided below.

## Methods

### FrACT Visual Acuity Version 3.10.5

The FrACT is a validated electronic software which was mainly developed for research purposes in order to obtain more precise acuity measurements (Bailey and Lovie-Kitchin, 2013). The acuity test optotypes are letters presented with a fixed number of trials (Bach and Schäfer, 2016).^1^ The presentation sequence follows an adaptive staircase method to determine threshold (Bach, 2006). Schulze-Bonsel et al. (2006) found that visual acuity results of ETDRS and FrACT closely agree.

The visual acuity of the child was measured binocularly while the participant was wearing their habitual distance correction. The caregiver was instructed to have a millimeter ruler and a tape measure to calibrate test distance for the FrACT Visual Acuity test. A printable millimeter ruler was attached to the invite email.^2^ To assess visual acuity we used the Landolt-C Acuity. Although the FrACT Landolt-C has eight possible orientations, a two-forced choice method Up/Down (vertical) was utilized. We eliminated orientations with Left/Right (horizontal) components due to recent experience during telehealth visits which found the vertical two-forced choice method to be more effective, efficient, and more likely to maintain patient interest than the four-choice paradigm.

### CVIT 3–6

The CVIT 3–6 is an objective assessment tool that uses a simple matching paradigm to assess the child’s visual perceptual abilities. We followed the same procedure guidelines provided by Vancleef et al. (2019). Although, the investigators performed this test in an in-person setting. This test was accessed by the examiner with the internet link^3^ and shared with the participant’s caregiver and the participant *via* Zoom for Telehealth screensharing. In order for the investigator to observe the child’s responses during testing, aside from the camera in the device the child was viewing, an auxiliary smart device (e.g., cellphone or tablet) was utilized positioned appropriately by the child’s caregiver for the examiner to have an additional view of the child during testing. The instruction given to the participant was to match an object with the object in a set of three alternatives. Any indication of a response was accepted: simple pointing to the matching object, tapping on the object on the screen, or a verbal response, or using the computer mouse. The examiner ensured that the participant responded to all test trials by friendly encouragement. Breaks were allowed after completion of the first domain and the number of breaks were recorded at the end the test. Scores were recorded for participants who successfully completed the test. The highest possible score is 70. Scores lower than 53 are considered below normal and possibly indicative of CVI. The CVIT 3–6 automatically calculates the overall score and constructs a graphical representation of scores across 14 subtests.

### The Flemish CVI Questionnaire

This is a validated questionnaire which consists of 46 binary closed-ended questions completed by the child’s caregiver (Ortibus et al., 2011a, b; Itzhak et al., 2020). The information from this survey is useful for characterizing behavioral difficulties, particularly regarding vision for action, vision while moving, and spatial orientation, which provides evidence for the possibility of CVI. The parent checks the most appropriate response for each item. The possible responses are: agree, disagree or NA (not applicable). Under normal circumstances, the questionnaire is available as a hard copy and is filled by the caregiver. Due to the virtual nature of this study, Redcap, a secure web forum for creating and managing online databases and surveys, was used to obtain a digital version of the questionnaire that can be accessed remotely.^4^ The finished online version of this questionnaire was reviewed and approved by Nofar Ben Itzhak, MSc and Els Ortibus, Ph.D. through personal communication.

### The Vineland-3 Comprehensive Parent/Caregiver Form

The Vineland-3 is a standardized measure of adaptive behavior, that is, the things that people do to function in their everyday lives (Sara et al., 2016). It is available in digital form which provides two delivery options: on-screen administration and remote-administration *via* email invites. The Vineland-3 is designed for mental health specialists, educators, and other professionals to use. It automatically generates a simple scoring and interpretive report eliminating the need for interpretation by certified/specialized personnel. Ability measures focus on what the examinee can do in a testing situation while the Vineland-3 focuses on what they actually do in daily life. The Domain Level tests four adaptive behavior domains, Communication, Daily Living Skills, Socialization, and Motor skills. Because it is a norm-based instrument, the examinee’s adaptive functioning is compared to that of others of their age. Age equivalent scores are derived from the subject’s measured raw scores in reference to the normative sample median. The electronic Vineland-3 was completed by the subject’s caregiver *via* a computer or a smartphone. Developmental age equivalence was derived from the Vineland-3 results to assign the participant to the appropriate age group. We utilized bootstrapping (a resampling method) around the median of the raw scores reported of the four adaptive behavior domains to obtain the developmental age.

## Results

Parents and caregivers of all participants were able to follow calibration instructions with ease. The FrACT visual acuity test was completed, instruction and explanation times included, in under 30 min. Table 1 shows individual acuity levels in the CVI and non-CVI groups respectively. Reported acuity levels are those obtained *via* the FrACT during the study and previously measured visual acuity obtained *via* reviewing the participant’s medical file.

**TABLE 1:** Summary of participants medical history and clinical characteristics.

Subject	VA on file (logMAR)	VA type	FrACT VA (logMAR)	Diagnoses and medical history	Ocular history
1	0.42	Symbol acuity*	1.2	CVI, Tuner syndrome, foveal hypoplasia, premature birth	Pendular nystagmus, derivational amblyopia
2	0.30	Symbol acuity	0.15	CVI, borderline microcephaly, hypotonia, full-term birth, global developmental delay	Left intermittent exotropia
3	0.40	Symbol acuity	0.88	CVI, FOXP1 syndrome, autism, PVL, developmental delay	Nystagmus, high hyperopia, astigmatism, partially accommodative strabismus
4	0.30	Letter acuity**	0	CVI, presumed *in utero* stroke, motor and language delay	Right visual field neglect
5	0.18	Symbol acuity	0.15	Full-term birth, Autism	Anisometropic hyperopia
6	0.18	Symbol acuity	0.04	Unremarkable, full-term birth	Exophoria
7	0.18	Symbol acuity	0.18	Left unicoronal craniosynostosis, speech delay	Anisometropic astigmatism, amblyopia

*Symbol acuity: Lea symbols. **Letter acuity: Snellen E.

Summary of data collected in the three assessment tools (CVIT 3–6, Flemish questionnaire, and the Vineland-3) are provided in Table 2. Results of the Vineland-3 are shown as age equivalent values in months. Chronological age is also shown on the table to give the reader an idea of the discrepancy between chronological age and age equivalent in the two groups. One participant in the CVI group could not completed the CVIT 3–6 as tasks became more difficult (indicated as NA in Table 2).

**TABLE 2:** Summary of collected data in all participants.

Subject	Flemish percent abnormal (%)	CVIT 3–6 score	Age equivalent (months)	Chronological age (months)	Diagnosis
1	8.66	42	73	40	CVI
2	11.36	65	39	61	CVI
3	12.87	NA	21	67	CVI
4	6.28	61	66	69	CVI
5	1.88	68	75	91	Non-CVI
6	0	62	52	49	Non-CVI
7	0	64	30	57	Non-CVI

## Discussion

Results of our small n study support the feasibility of adapting virtual models in data collection for various pediatric eyecare research projects. The FrACT visual acuity test calibrates for both screen size and distance from screen. In this project, the caregiver provided the measurements of both the calibration line (for screen size) and the distance from the screen. The researcher then entered that information into the settings screen prior to testing the child, thereby ensuring measurement accuracy. While the FrACT acuity system was validated for in office assessment (Bach, 2006), its use for remote assessment was not evaluated. In Table 1 we show VA levels of participants as indicated on their medical file vs. on the FrACT. However, it is worth noting that VA levels on file do not serve as a control since some were updated years ago and/or used symbol charts and not optotypes. For the future, we recommend a validation study of the use of the FrACT in this manner.

Although the CVIT 3–6 was performed remotely, it closely followed the same procedure suggested by Vancleef et al. (2019) with only one exception which is the child was observed *via* cameras. That said, the child’s attention may impact data collection. Since the child is in a familiar environment during their regular days, they might express less interest in following the clinician and in this case the investigator’s instructions. Especially that a digital screen is the sole means of communication. Environmental distractors included the use of toys or food (e.g., candy) by the caregiver as encouragement for the child during the test. Furthermore, technical issues can slow down the process which could negatively impact the child’s interest.

The Vineland-3 already had the option for remote asynchronous data collection. Its automatic generation of easily understood reports allow for this tool to be utilized by educators and professionals. Parents were encouraged to reach out to the principal investigator for any questions they might have filling out the questionnaire, particularly since the Vineland-3 is a lengthier and more comprehensive questionnaire. In the trial study which included mostly children of the faculty at New England College of Optometry, one parent reached out for clarification regarding the questionnaire. However, none of the caregivers in the CVI study had done so. This is one limitation of remote administration of questionnaires as it is expected that caregivers are less motivated to ask questions when completion the Vineland-3 without immediate access to the research.

We piloted the virtual version of this project during the summer of 2020. Patient recruitment occurred in the fall and early winter of 2020 and finally in January 2021. The low response to participation is postulated to be related to the combination of access to devices, stress of managing the household’s access and use of devices for school and work and otherwise caring for their children while being employed. It is anticipated as the pandemic wanes, there will be more flexibility to participate in remote studies, such as this one.

Remote testing is a cost-effective approach to improve timely access to care and early screening for CVI, especially in remote rural areas that might not have access to in-person care facilities. However, our study is limited by the low number of participants recruited. Furthermore, The adopted approach is limited to families who have access to digital devices and to a stable internet connection.

In conclusion, this study shows that virtual testing of young patients using complex tools is feasible in pediatric eye research. The model we adapted is more convenient for many families. In a broader perspective, such model can be scaled for larger studies of rare conditions, such as CVI without being confined by proximity to the researcher.

## Data Availability Statement

The original contributions presented in the study are included in the article/supplementary material, further inquiries can be directed to the corresponding author.

## Ethics Statement

The studies involving human participants were reviewed and approved by the NECO Institutional Review Board. Written informed consent to participate in this study was provided by the participants’ legal guardian/next of kin.

## Author Contributions

RA: design of the manuscript, writing, and editing. BK: writing and editing. Both authors contributed to the article and approved the submitted version.

## Conflict of Interest

The reviewer LL declared a past co-authorship with one of the authors BK to the handling Editor. The authors declare that the research was conducted in the absence of any commercial or financial relationships that could be construed as a potential conflict of interest.

## Publisher’s Note

All claims expressed in this article are solely those of the authors and do not necessarily represent those of their affiliated organizations, or those of the publisher, the editors and the reviewers. Any product that may be evaluated in this article, or claim that may be made by its manufacturer, is not guaranteed or endorsed by the publisher.

## Footnotes

1
https://michaelbach.de/fract/

2
https://www.readers.com/blog/wp-content/uploads/2016/07/Rea-PrintableSizeRuler.pdf

3
https://psytests.be/clinicians

4
https://redcap.neco.edu/redcap/surveys/?s=M9FXR9NC48
